# CMR ventriculometry for evaluation of ecg hypertrophy criteria in a preventive medicine population

**DOI:** 10.1186/1532-429X-13-S1-P244

**Published:** 2011-02-02

**Authors:** Holger C Eberle, Ulrike Stevka, Mani Farazandeh, Cristoph J Jensen, Thomas Schlosser, Kai Nassenstein, Christoph K Naber, Georg V Sabin, Oliver Bruder

**Affiliations:** 1 Elisabeth Hospital Essen, Essen, Germany; 2 University Hospital Essen, Essen, Germany

## Introduction

Left ventricular hypertrophy (LVH) is an important prognosticator for cardiovascular (CV) risk in persons with and without arterial hypertension. Given the high prevalence of these conditions, LVH screening is mandatory for CV prevention. Although not evaluated in a middle aged preventive medicine population, Electrocardiography (ECG) is widely used for LVH screening. Although not widely accessible as a screening technique, cardiovascular magnetic resonance (CMR) offers a unique opportunity to calculate LV mass index (LVMI) as the gold standard for other diagnostic tests.

## Purpose

To assess the reliability of ECG LVH criteria compared to CMR ventriculometry (cutoff: LVMI 83 g/m^2^) and the incremental value of CMR in a population from a prevention programme.

## Methods

220 (206 male, age 49.5 ± 8,4 years) consecutive participants of a prevention programme without known heart disease underwent CMR in a 1.5 T scanner (Magnetom Avanto, Siemens, Erlangen, Germany) including SSFP Cine (TrueFISP TR 3ms, TE 1.5 ms, FA 72°, slice thickness 6 mm) and delayed enhancement imaging.

LVMI was derived from contiguous short axis cine images using the summation of discs method. These data were compared to Sokolow-Lyon amplitude, Sokolow-Lyon duration product, Cornell amplitude, Cornell duration product, and Romhild-Estes score (Tables 1,2) in 12 lead ECG.

**Table 1:** Calculation of the ECG criteria

Criterion	Calculation	Normal value
Sokolow-Lyon Amplitude (mV)	SV1 + RV5/V6	<3,5
Sokolow-Lyon Duration Product (mm * ms)	(SV1 + RV5/V6) * QRS duration	<2940
Cornell Amplitude (mV)	R aVL + SV3	<2,8
Cornell Duration Product (mm + ms)	(R aVl + SV3) * QRS duration	<2440

**Table 2:** Romhild Estes Score

Criterion	Points
R or S > 20 mm or S in V1 or V2 > 30 mm or R in V5 or V6 30 mm	3
ST/T changes typical of LVH, taking digitalis	1
ST/T changes typical of LVH, not taking digitalis	3
P terminal force in V1 1mm or more, duration >40 ms	3
Left axis deviation > -30°	2
QRS dura> 90 ms	1
Intrinsicoid deflection in V5 or V6 >50 ms	1

## Results

**Figure 1:**
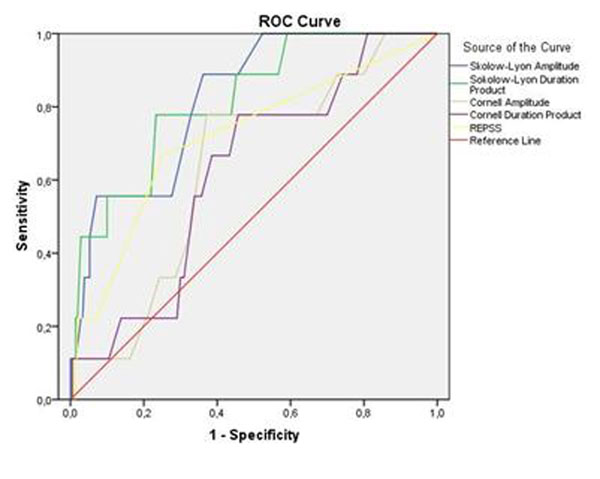
ROC analysis of the ECG criteria to identify LVH

**Figure 2:**
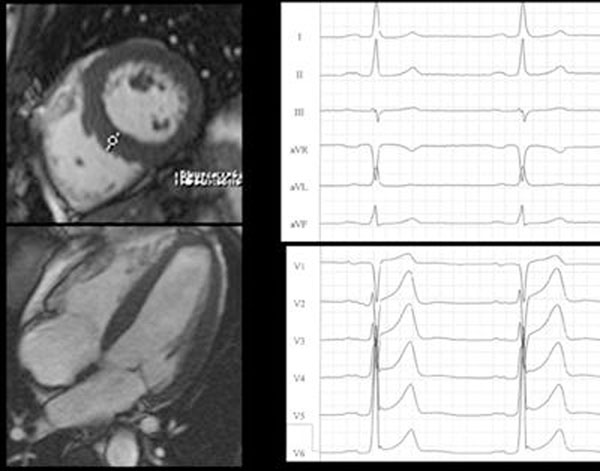
CMR showing LVH with increased LVMI and septal thickness, in a patient with pathologic Sokolow-Lyon Index, Sokolow Duration Product, and REPSS.

31 participants had positive LVH criteria in ECG, of which 6, and 4 ECG negatives, showed LVH (LVMI > 83 g/m^2^) in CMR (Figure 2). All ECG criteria were positively correlated with LV mass (Table 3). Specificities and negative predictive values were high in our low-prevalence population, but sensitivities and positive predictive values were poor (Table 3, Figure 1).

**Table 3:** Diagnostic characteristics and correlation with LVMI of the ECG criteria

Criterion	Correlation Coefficient (Perason)	Sensitivity	Specificity	Posisitive Predictive Value	Negative Predictive Value
Sokolow-Lyon Amplitude	0.413	0.52	0.94	0.26	0.98
Sokolow-Lyon Product	0.436	0.44	0.92	0.18	0.92
Cornell Amplitude	0.224	0.111	0.97	0.14	0.95
Cornell Product	0.231	0.2	0.97	0.11	0.96
REPSS	0.248	0.22	0.93	0.12	0.97

## Conclusions

CMR identified a large proportion of false positive ECG results (25/31), and identified additional LVH cases (4/189). ECG is not a reliable LVH screening tool in a middle aged prevention population. LVH screening should be based on imaging techniques.

